# Whole genome sequencing of *Neolamarckia macrophylla* (Roxb.) Bosser and *Neolamarckia cadamba* (Roxb.) Bosser from Indonesia: a vital resource for completing chloroplast genomes and mining microsatellite markers

**DOI:** 10.3389/fpls.2025.1608577

**Published:** 2025-06-20

**Authors:** Fifi Gus Dwiyanti, Irsyad Kamal, Rahadian Pratama, Dhika Syaputra, Evayusvita Rustam, Ratna Uli Damayanti, Iskandar Z. Siregar, Dede J. Sudrajat

**Affiliations:** ^1^ Department of Silviculture, Faculty of Forestry and Environment, Institut Pertanian Bogor (IPB University), Bogor, West Java, Indonesia; ^2^ Molecular Science Research Group, Advanced Research Laboratory, Institut Pertanian Bogor (IPB University), Bogor, West Java, Indonesia; ^3^ Department of Biochemistry, Faculty of Mathematics and Natural Sciences, Institut Pertanian Bogor (IPB University), Bogor, West Java, Indonesia; ^4^ Research Centre for Applied Botany, National Research and Innovation Agency/Badan Riset dan Inovasi Nasional (BRIN), Cibinong, Bogor, West Java, Indonesia

**Keywords:** chloroplast, genome, illumina, microsatellite, *Neolamarckia*, plant breeding

## 1 Introduction

The *Neolamarckia cadamba* (Roxb.) Bosser, commonly known as white jabon, and *Neolamarckia macrophylla* (Roxb.) Bosser, referred to as red jabon, are fast-growing tree species native to Indonesia, belonging to the Rubiaceae family. In recent years, these trees have drawn significant attention for their versatile applications in industrial plantations, community forests, and projects focused on forest and land rehabilitation (Kallio et al., 2011; Irawan and Purwanto, 2014; Sarjono et al., 2017). Both *N. cadamba* and *N. macrophylla* are used for various purposes, including the production of wood for the pulp industry, plywood manufacturing, and construction and carpentry materials (Soerianegara and Lemmens, 1993; Kartawinata, 1994; Lempang, 2014). Additionally, *N. cadamba* is recognized for its potential medicinal benefits, including pain relief, anti-inflammatory properties, antipyretic effects (Mondal et al., 2009), as well as antimicrobial (Acharyya et al., 2011), and antibacterial (Mishra and Siddique, 2011) activities.

The cultivation of *N. cadamba* and *N. macrophylla* is still hindered by the lack of access to superior seeds from breeding programs. Most seeds used for various planting initiatives are sourced from natural forests or plantation forests that are classified as Identified Seed Stands (TBT), where no tree breeding activities have been conducted (UPT Perbenihan Tanaman Hutan Jawa Timur, 2022). As a result, these stands often exhibit high growth variability, along with low productivity and poor wood quality. To address these issues, prioritizing the development of fast-growing native species should be a focus for tree breeding and silviculture research. This approach can help create superior native forest plant species that could potentially reduce the dominance of exotic species in virtually all industrial plantation forests across Indonesia. However, there is still limited genetic information available for *N. cadamba* and *N. macrophylla* from Indonesia, particularly concerning genome sequencing and microsatellite markers, also known as Simple Sequence Repeats (SSRs). This lack of data poses challenges for developing effective cultivation strategies, as these data are needed for genetic research and breeding.

Chloroplast genomes are valuable for phylogenetic analysis because they are predominantly maternally inherited, possess a conserved gene structure and content, and exhibit a low mutation frequency (Palmer et al., 1988). Moreover, chloroplast genomes provide essential data for population genetics, molecular identification, and genetic engineering (Powell et al., 1995; Daniell et al., 2016; Cao et al., 2022). On the other hand, microsatellite markers play crucial role in cultivar identification, assessing genetic diversity, genome mapping, quantitative trait loci (QTL) analysis, paternity analysis, cross-species transferability, segregation analysis, phylogenetic relationships, and identification of wild cross hybrids in plant species (Miah et al., 2013; Ahmad et al., 2018).

To date, several studies have focused on whole-genome sequencing of two species. One study characterized the complete genome of *Neolamarckia macrophylla* from South Sulawesi, Indonesia, using the Illumina HiSeq Nova platform and examined its phylogenetic relationship with *N. cadamba* and other species (Shi et al., 2020). Another study characterized the complete chloroplast genome of *Neolamarckia cadamba* from Guangdong province of China using Illumina pair-end sequencing (Li et al., 2018). However, both studies lack the development of microsatellite markers. This gap highlights the critical need to investigate the genomes and microsatellites of *N. cadamba* and *N. macrophylla* from Indonesia, as these populations may harbor unique genetic characteristics. In this study, whole genome sequencing data from *N. cadamba* and *N. macrophylla* were used to complete the chloroplast genome and screen microsatellite (or Simple Sequence Repeats, SSRs) markers for future molecular studies.

### 1.1 Plant material, DNA extraction, and sequencing

The plant material used in this study consisted of silica-gel dried leaf samples collected from the adult tree of *Neolamarckia cadamba* (white jabon) planted in Kediri Forest Management Unit (KPH), Kediri Regency, East Java Province, Indonesia (-7°55’38,61163” S, 112°11’46,13546” E) and the adult tree of *Neolamarckia macrophylla* (red jabon) planted in Special Purpose Forest Area (KHDTK) Parung Panjang, Bogor Regency, West Java Province, Indonesia (-6°23’9,54262” S, 106°31’23,97738” E). The *Neolamarckia cadamba* tree used in this study originated from Nusakambangan Island, Indonesia (Sample code: AI NJ18), while the *Neolamarckia macrophylla* tree was sourced from Laeya District, South Konawe Regency, Southeast Sulawesi Province, Indonesia (Sample code: S016–1 Prov1 Blok3). The silica-gel dried leaf samples from the two species underwent genomic DNA extraction using the Cetyltrimethylammonium bromide (CTAB) method (Doyle and Doyle, 1990). Initial quantification and purity of the genomic DNA were observed using Nanodrop 2000 (Thermo Scientific) and visualized through agarose gel electrophoresis. Qubit dsDNA BR Assay Kits (Thermo Scientific) were used for accurate DNA quantification. The genomic DNA of *N. cadamba* (concentration of 120 ng/μL and amount of 2,23 μg) and *N. macrophylla* (concentration of 7,96 ng/μL and amount of 0,398 μg) that passed the quality check were then subjected to library preparation and whole genome sequencing utilizing the Illumina NextSeq 500 System, producing a data output of 6 GB per sample.

### 1.2 Chloroplast genome assembly and annotation

Sequencing data were uploaded to the Galaxy web platform, specifically the public server at usegalaxy.org version 23.1.2.dev0 (https://usegalaxy.eu/) for analysis (Afgan et al., 2016). The quality of raw reads was assessed using FASTQC version 0.12.1 (https://www.bioinformatics.babraham.ac.uk/projects/fastqc/) (Andrews, 2010), and clean reads were filtered with Fastp version 0.23.2 (https://github.com/OpenGene/fastp) (Chen et al., 2018) using the default parameters. Clean reads were assembled using SPAdes version 3.15.3 (Bankevich et al., 2012) and NOVOPlasty version 4.3.1 (Dierckxsens et al., 2017), both with default parameters. The assembly results were then annotated using GeSeq (https://chlorobox.mpimp-golm.mpg.de/geseq.html) (Tillich et al., 2017). The fully annotated genome was illustrated using OrganellarGenomeDRAW v1.3.1 (Greiner et al., 2019).

### 1.3 Microsatellite marker

Microsatellite (SSR) markers were extracted using Krait: Microsatellites Investigation and Primer Design version v1.5.1 (Du et al., 2018) from the scaffolds of *N. cadamba* and *N. macrophylla*. The minimum repetition rates were set as follows: six for motifs with two bases and five for motifs with three, four, five, and six bases. A minimum gap of 100 bases was maintained between different microsatellite motifs. Sequences containing these microsatellite motifs were selected based on two criteria: (i) the flanking regions must be at least 150 base pairs (bp) in length on both sides and (ii) the microsatellite repeats should have the longest repeat motif. The microsatellite markers were then designed using Krait: Microsatellites Investigation and Primer Design, version 1.5.1 (Du et al., 2018), with default parameters.

## 2 Results

**Figure 1:**
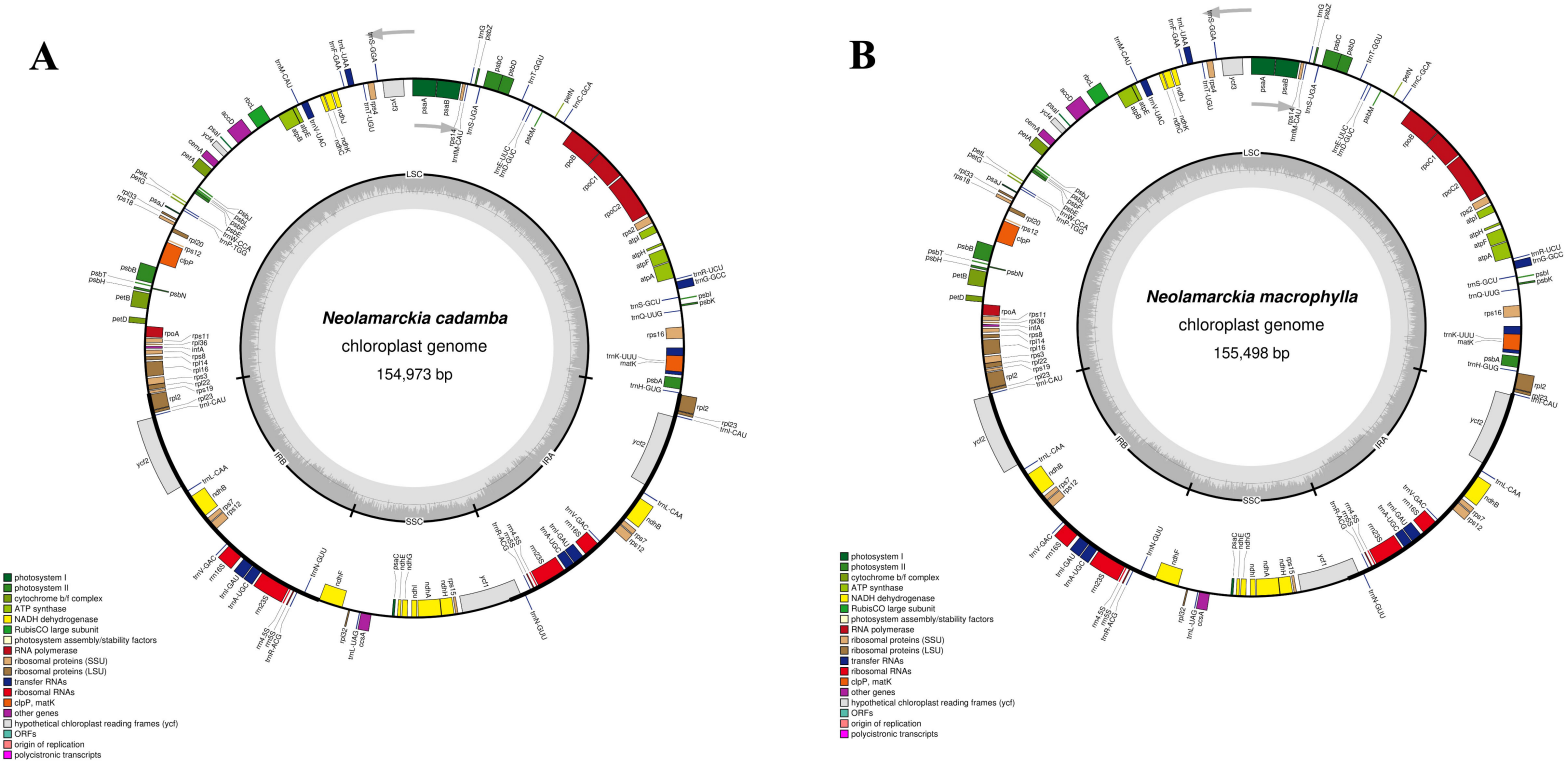
The complete chloroplast genomes of the two *Neolamarckia* species: **(A)** *Neolamarckia cadamba* and **(B)** *Neolamarckia macrophylla*.

The chloroplast genome of *N. cadamba* exhibited a typical quadripartite structure (**Figure 1**) with a total length of 154,973 bp. This genome comprises small single copy (SSC: 17,845 bp) and large single copy (LSC: 85,861 bp) regions, separated by a pair of inverted repeat regions: inverted repeat A (IRA: 25.633 bp) and inverted repeat B (IRB: 25,634 bp) (**Figure 1**). The *N. cadamba* chloroplast genome contained 126 genes in total, including 84 protein-coding genes (78 of which are unique), 36 transfer RNA (tRNA) genes (29 unique), and 8 ribosomal RNA (rRNA) genes, comprising 4 unique rRNA sequences (**Supplementary Table 1**). The GC content of the *N. cadamba* sequence is 37.6% (LSC: 35.4%; SSC: 31.6%; IR: 43.2%). These findings are consistent with previous results reported by Li et al. (2018), that the size of the chloroplast genome of *N. cadamba* was 154,999 bp, harboring an SSC of 17,851 bp, LSC of 85,880 bp, and a pair of inverted repeats (IRs) of 25,634 bp. Li et al. (2018) also reported the presence of 130 genes, where 96 were unique and 17 were duplicated in the IRs. The coding regions comprised 79 protein genes, 30 tRNA genes, and 4 rRNA genes, and the overall GC content of the chloroplast genome was 37.6%.

Similarly, the chloroplast genome of *N. macrophylla* also displays a quadripartite structure (**Figure 1**) with a length of 155,498 bp. This genome includes small single copy (SSC: 18,100 bp) and large single copy (LSC: 88,847 bp) regions, similarly separated by inverted repeat regions: IRA (24,275 bp) and IRB (24,276 bp) (**Figure 1**). The *N. macrophylla* chloroplast genome consisted of 126 genes, encompassing 84 protein-coding sequences with 29 unique sequences, 36 transfer RNA (tRNA) genes (29 unique), and 8 ribosomal RNA (rRNA) genes comprising 4 unique rRNA sequences (**Supplementary Table 1**). The GC content of the *N. macrophylla* sequence is 37.5% (LSC: 35.6%; SSC: 31.6%; IR: 43.4%). These findings align with the study by Shi et al. (2020), which revealed that the complete chloroplast genome of *N. macrophylla* is 155,406 bp and includes an SSC of 18,063 bp, an LSC of 86,013 bp, and a pair of IR regions of 25,665 bp each. Shi et al. (2020) also identified 128 genes, including 8 rRNA, 36 tRNA, and 84 protein-coding genes, and reported that the overall GC content of the chloroplast genome was 37.56%.

The analysis of microsatellites (SSRs) in *N. macrophylla and N. cadamba* (**Table 1**) revealed that *N. macrophylla* contained a greater number of SSRs, totaling 157,972, compared to 112,439 for *N. cadamba*. Additionally, the number of sequences containing SSRs was also greater in *N. macrophylla*, with 50,133 sequences compared to 34,884 in *N. cadamba*. Furthermore, candidate microsatellite markers were selected based on the richness of T/C content, consisting of 20 markers for *N. cadamba* (**Supplementary Table 2**) and 20 markers for *N. macrophylla* (**Supplementary Table 3**). The selected microsatellite marker candidates for each species will play a crucial role in future comprehensive analyses aimed at unraveling the genetic diversity of each species. This investigation will enhance the understanding of their unique genetic structures and evolutionary relationships, thereby providing invaluable support for advanced breeding programs.

**Table 1:** Summary of identified microsatellites (SSRs) from the genome assembly of *Neolamarckia cadamba* and *Neolamarckia macrophylla*.

Features	Number	
	*Neolamarckia macrophylla*	*Neolamarckia cadamba*
Total number of identified SSRs	157,972	112,439
Number of SSR-containing sequences	50,133	34,884
Number of sequences containing more than 1 SSR	49,078	43,391
Number of SSRs present in compound formation	12,551	9,522
**Motifs:**		
Dinucleotide	136,850	97,038
Trinucleotide	14,040	9,912
Tetranucleotide	5,500	4,634
Pentanucleotide	718	363
Hexanucleotide	759	449

## Data Availability

The datasets presented in this study can be found in online repositories. The names of the repository/repositories and accession number(s) can be found in the DNA Data Bank of Japan (DDBJ) under the Bioproject number PRJDB20739 (https://ddbj.nig.ac.jp/search/entry/bioproject/PRJDB20739). The Bio-sample number for *Neolamarckia cadamba* (white jabon) is SAMD00911405 (https://ddbj.nig.ac.jp/search/entry/biosample/SAMD00911405), and the Bio-sample number for *Neolamarckia macrophylla* (red jabon) is SAMD00911406 (https://ddbj.nig.ac.jp/search/entry/biosample/SAMD00911406).
