# Supplementary material for: Whole genome sequencing of Neolamarckia macrophylla (Roxb.) Bosser and Neolamarckia cadamba (Roxb.) Bosser from Indonesia: a vital resource for completing chloroplast genomes and mining microsatellite markers
Source: Front Plant Sci. 2025 Jun 20;16:1608577. doi: 10.3389/fpls.2025.1608577 (PMC12227011; doi:10.3389/fpls.2025.1608577)
Supplement: Supplementary file 1 [file Table1.docx]

Supplementary Material

# Supplementary Tables

## Tables

**Supplementary Table 1.**List of genes in the chloroplast genomes of *Neolamarckia cadamba* and *Neolamarckia macrophylla*

| Functional category | Group of Gene | Names of genes for  *Neolamarckia cadamba* | Names of genes for  *Neolamarckia macrophylla* |
| --- | --- | --- | --- |
| Self-replication | rRNA | *rrn*16^d^, *rrn*23^d^, *rrn*4.5^d^, *rrn*5^d^ | *rrn*16^d^, *rrn*23^d^, *rrn*4.5^d^, *rrn*5^d^ |
|  | tRNA | *trn*K-UUU*, *trn*Q-UUG, *trn*S-GCU, *trn*S-GGA, *trn*R-UCU, *trn*C-GCA, *trn*D-GUC, *trn*Y-GUA, *trn*E-UUC, *trn*T-GGU, *trn*S-UGA, *trn*GGCC, *trn*M-CAU, *trn*T-UGU, *trn*L-UAA*, *trn*F-GAA, *trn*fMCAU, *trn*W-CCA, *trn*G-UCC*, *trn*P-UGG, *trn*H-GUG, *trn*L-CAA^d^, *trn*V-GAC^d^, *trn*V-UAC*, *trn*I-GAU^d^*, *trn*A-UGC^d^*, *trn*R-ACG^d^, *trn*N-GUU^d^, *trn*L-UAG, *trn*I-CAU^d^ | *trn*A-UGC^d^, *trn*C-GCA, *trn*D-GUC, *trn*E-UUC, *trn*F-GAA, *trn*fM-CAU, *trn*G-GCC*, *trn*H-GUG, *trn*I-GAU^d^*, *trn*I-CAU^d^, *trn*K-UUU*, *trn*L-CAA^d^, *trn*L-UAA*, *trn*L-UAG, *trn*M-CAU, *trn*N-GUU^d^, *trn*P-UGG, *trn*Q-UUG, *trn*R-ACG^d^, *trn*R-UCU, *trn*S-GCU, *trn*S-GGA, *trn*S-UGA, *trn*T-GGU, *trn*T-UGU, *trn*V-GAC^d^, *trn*V-UAC*, *trn*W-CCA, *trn*Y-GUA |
|  | Large subunit ribosomal proteins (LSU) | *rpl*14, *rpl*16*, *rpl*2^d^*, *rpl*20, *rpl*22, *rpl*23d, *rpl*32, *rpl*33, *rpl*36 | *rpl*14, *rpl*16*, *rpl*2^d^*, *rpl2*0, *rpl*22, *rpl*23d, *rpl*32, *rpl*33, *rpl*36 |
|  | Smallsubunit ribosomal proteins (SSU) | *rps*11, *rps*12d**, *rps*14, *rps*15, rps16*, *rps*18, *rps*19, *rps*2, *rps*3, *rps*4, *rps*7^d^, *rps*8 | *rps*11, *rps*12d**, *rps*14, *rps*15, rps16*, *rps*18, *rps*19, *rps*2, *rps*3, *rps*4, *rps*7^d^, *rps*8 |
|  | DNA-dependent RNA polymerase | *rpo*A, *rpo*B, *rpo*C1*, *rpo*C2 | *rpo*A, *rpo*B, *rpo*C1*, *rpo*C2 |
|  | Subunits of ATP synthase | *atp*A, *atp*B, *atp*E, *atp*F*, *atp*H, *atp*I | *atp*A, *atp*B, *atp*E, *atp*F*, *atp*H, *atp*I |
|  | Subunits of NADH-dehydrogenase | *ndh*A*, *ndh*B^d^*, *ndh*C, *ndh*D, *ndh*E, *ndh*F, *ndh*G, *ndh*H, *ndh*I, *ndh*J, *ndh*K | *ndh*A, *ndh*B^d^*, *ndh*C, *ndh*D, *ndh*E, *ndh*F, *ndh*G, *ndh*H, *ndh*I, *ndh*J, *ndh*K |
| Photosynthesis | Subunits of photosystem I | *psa*A, *psa*B, *psa*C, *psa*I, *psa*J | *psa*A, *psa*B, *psa*C, *psa*I, *psa*J |
|  | Subunits of photosystem II | *psb*A, *psb*B, *psb*C, *psb*D, *psb*E, *psb*F, *psb*H, *psb*I, *psb*J, *psb*K, *psb*L, *psb*M, *psb*T, *psb*Z, ycf1, *ycf*2^d^ | *psb*A, *psb*B, *psb*C, psbD, *psb*E, *psb*F, *psb*H, *psb*I, *psb*J, *psb*K, *psb*L, *psb*M, *psb*T, *psb*Z, *ycf*1,*ycf*2^d^ |
|  | Subunits of cytochrome b/f complex | *pet*A, *pet*B*, *pet*D*, *pet*G, *pet*L, *pet*N | *pet*A, *pet*B*, *pet*D*, *pet*G, *pet*L, *pet*N |
|  | Subunit rubisco | *rbc*L | *rbc*L |
|  | Subunit of acetyl-CoA-carboxylase | *acc*D | *acc*D |
|  | C-type cytochrome synthesis gene | *ccs*A | *ccs*A |
| Other function | Protease | *clp*P1** | *clp*P1** |
|  | Maturase | *mat*K | *mat*K |
|  | Translation Initiation Factor IF-1 | *inf*A | *inf*A |
|  | IPhotosystem assembly factors | *paf*II, *paf*I* | *paf*II, *paf*I* |
|  | Photosystem biogenesis factor | *pbf*1 | *pbf*1 |
|  | Envelope membrane protein | *cem*A | *cem*A |
| Unknown function | Conserved open reading frames | *ycf*1^d^, *ycf*2^d^ | *ycf*1, *ycf*2^d^ |

(d) gene duplications, (*) single intron, (**) double intron.

**Supplementary Table 2.** Selected 20 microsatellite (SSR) markers for *Neolamarckia cadamba*

| ID | Sequence | Motif | Forward | Reverse |
| --- | --- | --- | --- | --- |
| 1 | NODE_1913 | TC(39) | CAATCCGGTCCATGAAAGG | GGAAGAGGATTAGGGGAGGC |
| 2 | NODE_56845 | TATCTA(13) | ACCTTGGGTCATTTGGTTGG | ATGCAAAGACTCTAAAACTCACC |
| 3 | NODE_319 | AG(38) | TTCAGACCCCACAACCTAGG | AAACAGGGAAGATGGAGGGG |
| 4 | NODE_1380 | ACAT(19) | ATGCCCAATGCCTAAACAGC | AAAAGTACAGTCACCAAGGGG |
| 5 | NODE_5638 | AAG(25) | TGATAAAGCCCAGAGACAGC | TCCTGAGAATCCGAGGATGC |
| 6 | NODE_10962 | ATT(25) | TAAACCACAAGGCACACAGC | GCACAAAGAACTCTTCACGC |
| 7 | NODE_1323 | TC(37) | TTCTTAGGAATCTTCTTCTCCGG | TCTTGGGTTCACCTACTGGG |
| 8 | NODE_128 | TTA(24) | GAAACCCGTTCAAAACTCGC | AATTCTAATGCGTCACGTAACG |
| 9 | NODE_2731 | ATT(24) | AGTGAAGCATGAACTGTGGC | GGAAGGCTGCTTGTATGAGG |
| 10 | NODE_3183 | CT(36) | GTTAGTTGCTCTTCTCGCGC | AACCCCCGTCTACTATTGGG |
| 11 | NODE_3365 | CT(36) | GGGCCCGTGAACAAATTCG | TCTGGTTCACTAATTTCCGATGC |
| 12 | NODE_8277 | ATA(24) | TGTTTGAGGTGCAAGGTAAGC | CCCTTGGGAAGAGAACAACG |
| 13 | NODE_54453 | TAA(24) | GTAAGGGGTTTGGCTTTCCC | ATCACCCCCAGATGATGTCC |
| 14 | NODE_71234 | AG(36) | TGTCCATTTTCGAGAAGTCACC | TGCTTCCTGATAGTTTCTTCGC |
| 15 | NODE_960 | CT(35) | CGTTTCTGCGTCTGACTGG | CTAGGGCTTCTTCATCACGC |
| 16 | NODE_1380 | CT(35) | GACCACCAGAGACTCAGAG | CCCTATCGTGAACATCCTGC |
| 17 | NODE_7177 | TC(35) | GAAGATCCCCTTCCTTCTTTCC | AACTCCCCTAACTACGGACC |
| 18 | NODE_20256 | CT(35) | CGGTTGAATTGTGCATGTGC | ACAAAGAGCTAGGGCCATGG |
| 19 | NODE_24038 | CT(35) | ACTTCCCATGCTTCCTATGC | CACAAGGCCAATTCAACTTGC |
| 20 | NODE_30236 | TC(35) | TTTCAGCTGCAAACCTGTCC | AACAACTTTCGCTTGCATGC |

**Supplementary Table 3.** Selected 20 microsatellite (SSR) markers for *Neolamarckia macrophylla*

| ID | Sequence | Motif | Forward | Reverse |
| --- | --- | --- | --- | --- |
| 1 | NODE_99720 | GAAA | CCCGATAACACACAGTTCGG | ACAAACACATCTTCCCAGCC |
| 2 | NODE_59856 | TA946) | TTTTCCCCGGTTTTGATATGC | GACAAGCCAAAGAAGGGTGG |
| 3 | NODE_427444 | TTTCG | GTTTCGTTCCGTTTCGTTTCG | ACGAAAAGAAACGAAACGAAACG |
| 4 | NODE_4602 | AT(430 | TAGGATGGAGAGTTGGGAGC | AGAGCTGACCCAAAAGAAGG |
| 5 | NODE_14509 | TAT(26) | GTTGTGATGTTCAATTTGGCCC | AATAAGGCCATCCAATCGCC |
| 6 | NODE_16602 | AT (39) | ACTCTGGCTAGATGAATGCG | TCAAAGTCACAAATTTGGCATCC |
| 7 | NODE_21136 | TC (39) | ACCCATTCCAATTAGCAGCC | ACGAGTAGAGCTGTGTATCGG |
| 8 | NODE_6186 | TC(38) | TCTTGCAATTCCGCGTATGC | GTTGTTACCCTTGCCACTGG |
| 9 | NODE_238 | TTC(25) | TTGTAGCACAACATTTGAAGGG | AGCTAAGGGAAGACTGGACG |
| 10 | NODE_3623 | AT(36) | GTGCAGAACTCCTTGGTAGC | TAACCTCAATGAGCTCGAGC |
| 11 | NODE_4032 | GA(36) | CCTTGAGTTCACGCATTTTAGC | AAAAGCAAAACGTGATGGCG |
| 12 | NODE_5790 | AG(36) | CAAGGTTGCGGATAGAGTGC | TTTGGCTAGCCATCCATTCC |
| 13 | NODE_9946 | TC(36) | TCAACCCCCAGTTACTCAGC | TGGTCAGAGTAGTTGATAAGCG |
| 14 | NODE_34989 | TA(36) | AGGGCACATTGAGGGATAGG | ATTGTAGGTTGCTCCACTGC |
| 15 | NODE_58583 | CT(36) | CCACTACGTGCGTATTCGG | AGGGCAAAAACCAATGACCC |
| 16 | NODE_87460 | TA(36) | AGTTTGCAAAGGGAAGCTGG | GCCCCTACATGCTCTTGC |
| 17 | NODE_240488 | CTCCAG(12) | GGCGAGCACTTCATCATCC | AGAGCTGGAAGAAGAGCTGG |
| 18 | NODE_128 | CT(35) | GGTAACGCAAGAGCAACACC | GCTCTAATTTCACTCCTCACTCC |
| 19 | NODE_3150 | AT(35) | ACAATTTCTACGCTTCTCTGCC | TCATCACCAGCTTGTCCTCC |
| 20 | NODE_4821 | TC(35) | GAATCCGTGACAGTAGTGCC | GATGAACATGTTGCACAGGC |
